# Systematic Screens for Proteins That Interact with the Mucolipidosis Type IV Protein TRPML1

**DOI:** 10.1371/journal.pone.0056780

**Published:** 2013-02-13

**Authors:** Ellen Spooner, Brooke M. McLaughlin, Talya Lepow, Tyler A. Durns, Justin Randall, Cameron Upchurch, Katherine Miller, Erin M. Campbell, Hanna Fares

**Affiliations:** Department of Molecular and Cellular Biology, University of Arizona, Tucson, Arizona, United States of America; Université de Genève, Switzerland

## Abstract

Mucolipidosis type IV is a lysosomal storage disorder resulting from mutations in the *MCOLN1* gene, which encodes the endosomal/lysosomal Transient Receptor Potential channel protein mucolipin-1/TRPML1. Cells isolated from Mucolipidosis type IV patients and grown *in vitro* and in *in vivo* models of this disease both show several lysosome-associated defects. However, it is still unclear how TRPML1 regulates the transport steps implicated by these defects. Identifying proteins that associate with TRPML1 will facilitate the elucidation of its cellular and biochemical functions. We report here two saturation screens for proteins that interact with TRPML1: one that is based on immunoprecipitation/mass spectrometry and the other using a genetic yeast two-hybrid approach. From these screens, we identified largely non-overlapping proteins, which represent potential TRPML1-interactors., Using additional interaction assays on some of the potential interactors from each screen, we validated some proteins as candidate TRPML1 interactors In addition, our analysis indicates that each of the two screens not only identified some false-positive interactors, as expected from any screen, but also failed to uncover potential TRPML1 interactors. Future studies on the true interactors, first identified in these screens, will help elucidate the structure and function of protein complexes containing TRPML1.

## Introduction

Mucolipidosis type IV (MLIV) is a neurodegenerative lysosomal storage disorder that is characterized by severe psychomotor retardation, achlorhydria, and ophthalmological abnormalities that lead to blindness. Most tissues in MLIV patients show lysosomal defects, yet death primarily occurs in neurons [1], [2]. MLIV is caused by mutations in the *MCOLN1* gene, which encodes a transient receptor potential ion channel protein called mucolipin-1/TRPML1 [3], [4], [5]. The TRPML1 channel is permeable to cations and localizes primarily to late endosomes/lysosomes [6], [7], [8], [9], [10], [11], [12]. Over 20 different mutations in *MCOLN1* have been identified in MLIV patients, although two founder mutations account for ∼95% of all MLIV alleles and show a heterozygote frequency of 1:100 in the Ashkenazi Jewish population [13], [14]. There are two other homologues of TRPML1, TRPML2 and TRPML3, and the three TRPMLs homo- and hetero-multimerize [15], [16].

Studies on MLIV cells have identified many lysosomal-associated defects, including defects in transport to lysosomes [17], [18], [19], [20], in lysosomal degradation leading to accumulation of material [21], [22], in lysosomal exocytosis [23], in lipid transport from endosomes to the Golgi apparatus [11], [19], [24], in metal homeostasis [7], [8], in macroautophagy [25], [26], in chaperone-mediated autophagy [27], and in mitochondrial function [28]. Yet the question still remains: which transport steps that are defective in MLIV cells are normally directly regulated by TRPML1? For example, does TRPML1 directly regulate lipid transport from endosomes to the Golgi apparatus, or is this defect in the absence of TRPML1 indirect, perhaps due to altered late endosomes/lysosomes? Furthermore, how does TRPML1 regulate the different transport steps?

One approach to begin to answer questions about the biochemical functions of TRPML1 is to identify proteins that directly associate with TRPML1 and/or are found in a TRPML1-containing complex. The molecular identities of these interactors may immediately suggest testable mechanisms. Furthermore, interfering with the functions of these interactors may implicate specific transport steps that they regulate in association with TRPML1. Previous studies identified three classes of proteins that physically associate with TRPML1. First, Alix/Apoptosis-Linked Gene-2 (ALG-2) is a penta-EF hand protein that binds the amino terminus of TRPML1 [20]. Second, Lysosomal-Associated Protein Transmembrane (LAPTM)-4a, LAPTM-4b, and LAPTM-5 associate with TRPML1 on endosomes/lysosomes [29]. Third, TRPML1 is also thought to associate with the chaperones Hsc70 and Hsp40 and other members of the Chaperone-Mediated Autophagy complex. While the physiological significance of the first two class of interactions has yet to be elucidated, TRPML1 is thought to regulate Chaperone-Mediated Autophagy through its interactions with the third class of proteins [27].

In this study, we systematically screen for additional proteins that associate with TRPML1. We report the observations from two screens, one biochemical and the other genetic, that surprisingly yielded minimally overlapping lists of potential TRPML1 interactors. We use several additional assays to identify candidate TRPML1 interactors from a subset of these lists.

## Materials and Methods

### Strains

Murine RAW264.7 macrophages and HeLa cells (ATCC, Manassas, VA) were grown in Dulbecco's Modified Eagle Medium (DMEM) containing 2 mM Glutamax and supplemented with 10% Fetal Bovine Serum, 100 U/ml penicillin, and 100 µg/ml streptomycin (Invitrogen, Carlsbad, CA) at 37°C in 95% air at 5% carbon dioxide. RAW264.7 stable clones expressing GFP-TRPML1 were previously described and were grown in the same medium supplemented with 250 µg/ml G418 [19].

### Plasmids

The following plasmids were used in this study:

- pcDNA/V5-DEST: Gateway (GTWY) destination vector with CMV promoter to add V5 epitope to COOH-terminus for mammalian expression (Invitrogen).

- pcDNA3.1/nV5-DEST: GTWY destination vector with CMV promoter to add V5 epitope to NH_2_-terminus for mammalian expression (Invitrogen).

- pDest-C-TagRFP: GTWY destination vector with CMV promoter to add TagRFP(S158T) to COOH-terminus for mammalian expression (this study).

- pDest-N-TagRFP: GTWY destination vector with CMV promoter to add TagRFP(S158T) to NH_2_-terminus for mammalian expression (this study).

- pPR3-C-GTWY: pPR3-Cvector (Dualsystems, Switzerland) modified for GTWY cloning. Destination vector to add NubG to COOH-terminus for split-ubiquitin yeast two-hybrid (this study).

- pPR3-STE-GTWY: pPR3-STE vector (Dualsystems) modified for GTWY cloning. Destination vector to add NubG to COOH-terminus for split-ubiquitin yeast two-hybrid (this study).

- pPR3-N-GTWY: pPR3-N vector (Dualsystems) modified for GTWY cloning. Destination vector to add NubG to NH_2_-terminus for split-ubiquitin yeast two-hybrid (this study).

- pEGFP-C3: Mammalian, CMV promoter, expression plasmid for EGFP fusions (BD Biosciences, Billerica, MA).

- pHD300: Mouse *Mcoln1* cloned in frame with EGFP at its NH2-terminus in pEGFP-C3 [19].

- pHD407: Mouse *Mcoln1* cloned in frame with Cub-LexA-VP16 at its COOH-terminus in split-ubiquitin yeast two-hybrid plasmid pBT3-STE (Dualsystems; this study).

Additional split-ubiquitin yeast two-hybrid plasmids include the positive controls pFur4-NubI and pOst1-NubI and the negative controls pFur4-NubG and pOst1-NubG [30].

Plasmids expressing epitope-fused candidate proteins are shown in Table S1. Additional details regarding the construction of plasmids in this study are available upon request.

### GFP-TRPML1 Immunoprecipitation and Mass Spectrometry

To identify TRPML1-associated proteins, we immunoprecipitated GFP-TRPML1 (mouse) using bead-conjugated anti-GFP (MBL, Woburn, MA) from lysates of RAW264.7 macrophages stably expressing GFP-TRPML1 [19]. Lysis was done using Lysis Buffer (20 mM Tris pH 7.5, 150 mM NaCl, 1% NP40, 5 mM EDTA, 0.42 mg/ml sodium fluoride, 0.368 mg/ml sodium orthovanadate, 0.0121 mg/ml ammonium molybdate, 0.04 Complete protease inhibitors tablet/ml [Roche Diagnostics, Mannheim, Germany]) and washes were done using TNEN Buffer (same as Lysis Buffer but with 0.5% NP-40), as previously described [31]. We then identified proteins that co-immunoprecipitated with GFP-TRPML1 using MudPIT analysis [32], [33]. To reduce the identification of non-specific co-purifying proteins, we performed the same procedure on stable RAW264.7 clones expressing the integral membrane protein Derlin-1-GFP as a negative control [34]. Samples were subjected to Mass Spectrometry three times to identify >90% of the proteins in each of the samples. Proteins in each sample were considered positive if they had an identification probability greater than 90% using the Scaffold program [35], [36], [37]. Proteins that were identified in the GFP-TRPML1 sample but not in the Derlin-1-GFP sample were considered potential TRPML1-specific interactors.

GFP-TRPML1 and Derlin-1-GFP, lysate and immunoprecipitation samples, were also subjected to Western analysis to determine whether endogenous proteins immunoprecipitate preferentially with TRPML1 or Derlin-1. The primary antibodies used were Rabbit anti-GFP (Abcam, Cambridge, MA), Mouse anti-STOML1 (Abnova, Walnut, CA), Chicken anti-SNX2 (Abcam), and Rabbit anti-Destrin (Gene Tex, Irvine, CA).

### GFP-TRPML1 Immunoprecipitation and Western Analysis

HeLa cells were transfected with a plasmid expressing GFP (pEGFP-C3) or GFP-TRPML1, along with the plasmid expressing a candidate protein fused to the V5 epitope. 30 μl of the 800 μl lysates were kept for analysis of total protein levels; the rest of the sample was subjected to immunoprecipitation using bead-conjugated anti-GFP. Total and immunoprecipitated proteins were subjected to Western analysis; equal amounts of different samples were loaded in each lane. Each candidate interactor was tested at least twice to confirm the immunoprecipitation result. The primary antibodies used were Rabbit anti-GFP (Abcam) and Mouse anti-V5 (Abcam).

### Split Ubiquitin Yeast Two-Hybrid Analyses

Split-ubiquitin yeast two-hybrid assays were performed using the Dualsystems Biotech kit. Plasmids expressing TRPML1-Cub-LexA-VP16 and NubG or NubI-fusions were transformed into the yeast strain NMY51 [*MATa his3delta200 trp1-901 leu2-3,112 ade2 LYS2::(lexAop)4-HIS3 ura3::(lexAop)8-lacZ (lexAop)8-ADE2 GAL4*)] and selected on SD–leu –trp plates. Equal numbers of cells from each transformation were spotted on SD–leu –trp and –leu –trp –ade –his +1 mM 3-AT plates and incubated at 30°C. Growth was scored over the next four days.

For the yeast two-hybrid screens, the NMY51 yeast strain bearing a mouse TRPML1-Cub-LexA-VP16 expression plasmid was transformed with expression libraries for mouse cDNAs fused to NubG. The libraries used were a mouse heart X-NubG cDNA library (Dualsystems) and a mouse NubG-X cDNA library (generous gift of Igor Stagljar). Transformations were plated on SD–leu –trp plates to assess numbers screened and on SD–leu –trp –ade –his +1−5 mM 3-AT plates to identify candidate interactors. More than 10^6^ colonies were screened for each library. The NubG plasmid was isolated in *Escherichia coli* from each colony that grew on SD–leu –trp –ade –his +1−5 mM 3-AT plates and was retransformed into the NMY51 strain bearing an TRPML1-Cub-LexA-VP16 expressing plasmid to confirm the interaction. Once confirmed, each plasmid was sequenced to identify the cDNA/gene and to confirm that the open reading frame was in-frame with NubG (those that were not in frame were discarded).

### GFP/TagRFP Imaging

RAW264.7 macrophages stably expressing GFP-TRPML1 were transfected with plasmids expressing TagRFP(S158T) fused to candidate proteins. Cells were fixed for 1 hour in 1% formaldehyde/1XPBS, washed three times with 1XPBS, and mounted in Slowfade mounting medium (Invitrogen) on slides for viewing. Confocal images were taken with a Nikon PCM 2000, using HeNe 543 excitation for the red dye and argon 488 for the green dye.

### Immunofluorescence

RAW264.7 macrophages stably expressing GFP-TRPML1 were transfected with plasmids expressing V5 fused to candidate proteins. Immunofluorescence was carried out as previously described [19]. Primary antibodies used were Rabbit anti-GFP (Abcam) and Mouse anti-V5 (Abcam).

### Determining Co-Localization with GFP-TRPML1

The percent co-localization is defined as the number of GFP-TRPML1-stained structures that co-localized with TagRFP(S158T)-fused or V5-fused structures divided by the total number of GFP-TRPML1-stained structures in a section and multiplied by 100. The graphs show the average from sections of at least eight different cells, with at least twenty GFP-TRPML1-stained structures per cell.

## Results

### Identification of TRPML1 Interactors by Immunoprecipitation and Mass Spectrometry

Our first approach for identifying TRPML1 interactors was immunoprecipitation combined with Mass Spectrometry. We immunoprecipitated GFP-TRPML1 or Derlin-1-GFP (an integral membrane protein found in the endoplasmic reticulum and endosomes) from stably expressing RAW264.7 clones in the absence of Ca^2+^ and then used Mass Spectrometry to identify proteins in each immunoprecipitate [19], [34]. Proteins that co-immunoprecipitated with GFP-TRPML1 but not Derlin-1-GFP were considered potential TRPML1-specific interactors (Table S2). While this approach allowed us to eliminate many non-specific interactors, the complexity of each sample imposes some limits on this stringency by detection failures. For example, some proteins that we characterized as candidates may actually be non-specific interactors that escaped detection in the Derlin-1-GFP sample, and likewise, not all of the actual TRPML1 interactors may have been detected using this approach. We therefore decided to use a second technique, the Split-Ubiquitin Yeast Two-Hybrid (SU-YTH) assay, to also screen for TRPML1 interactors. We reasoned that this complementary approach would generate a second list of candidates that we could compare to the Immunoprecipitation/Mass Spectrometry list to identify strong candidate TRPML1 interactors.

### Identification of TRPML1 Interactors by Split-Ubiquitin Yeast Two-Hybrid Screens

The Split-Ubiquitin Yeast Two-Hybrid (SU-YTH) assay is a genetic method for *in vivo* detection of membrane-protein interactions that is based on the reconstitution of an ubiquitin molecule in *Saccharomyces cerevisiae* [30]. Because proteins are not targeted to the nucleus, this method allows for yeast two-hybrid analysis of full-length integral membrane proteins. We expressed a TRPML1-Cub-LexA-VP16 fusion protein in yeast and monitored its interaction with Fur4 (plasma membrane localized) Ost1 (endoplasmic reticulum localized). When these test proteins were fused to NubG, which reduces its affinity for Cub, no interaction was detected, as was the case for the unfused NubG control (see below). In contrast, TRPML1-Cub-LexA-VP16 interacted with both Fur4-NubI and Ost1-NubI fusions, as expected. Intriguingly, the TRPML1-Fur4 interaction was stronger than the TRPML1-Ost1 interaction, suggesting that more TRPML1-Cub-LexA-VP16 protein remains in the endoplasmic reticulum than is secreted to reach the plasma membrane [30]..

We then transformed NubG-fused mouse cDNA libraries into yeast expressing TRPML1-Cub-LexA-VP16 and assayed for growth on selective media. We identified several potential TRPML1 interactors, which included Lysosomal-Associated Protein Transmembrane 4B that was previously identified as a TRPML1 interactor (Table S3) [29]. However, there were only a few candidate proteins that were identified using both the Immunoprecipitation/Mass Spectrometry and the SU-YTH approaches (highlighted in blue, red, and green in Tables S2 and S3). These included the same glyceraldehyde 3-phosphate dehydrogenase, homologous though not identical cadherins that are encoded by different genes, and homologous though not the same sodium channel alpha subunit encoded by two different genes. SU-YTH may fail to detect *bona fide* interactors that are unable to associate with TRPML1 in yeast or may also yield false-positives that only associate in the context of this assay. We therefore carried out additional assays to probe the effectiveness of Immunoprecipitation/Mass Spectrometry and SU-YTH for the purpose of identifying TRPML1 interacting proteins.

### Strategy for Identifying Candidate TRPML1 Interactors

We chose seven proteins identified by Immunoprecipitation/Mass Spectrometry list and six proteins identified from the SU-YTH screen to validate as TRPML1 candidate interactors with additional assays (highlighted in yellow in Tables S2 and S3). In addition, because we had identified the small GTPases Rac2 and Cdc42 by Immunoprecipitation/Mass Spectrometry, we tested two other closely related family members, Rac1 and RhoG (Table S2). Furthermore, we tested two Phosphatidylinositol 4-Phosphate 5-Kinase type I-beta (P5KT1) homologous proteins that are encoded by different genes, BAA13031 on chromosome 3 (a truncated form encoding the first 366 amino acids of this protein was identified by the SU-YTH screen; Table S3) and NP_032872 on chromosome 19.

**Figure 1:**
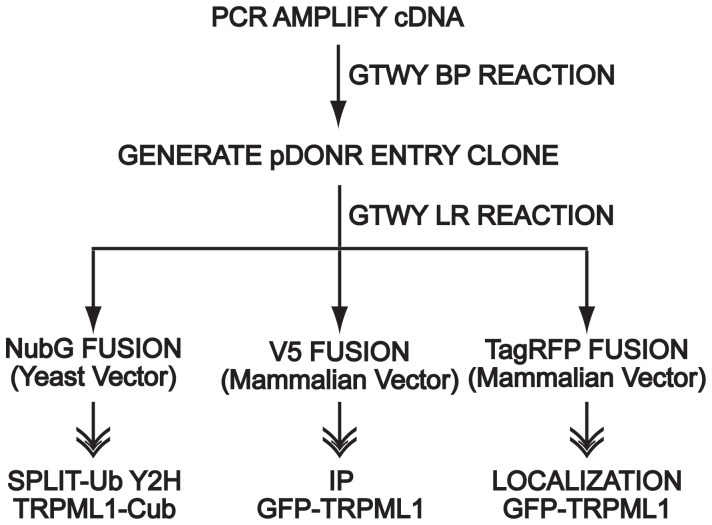
Cloning Strategy for Analyzing Candidate Interactors. Shown is a schematic of the GTWY cloning strategy for constructing the epitope-fused candidate proteins in the proper expression vectors.

We amplified by Polymerase Chain reaction (PCR) full-length mouse cDNAs corresponding to these proteins and used Gateway cloning to introduce these cDNAs into an ENTRY clone. We then used Gateway cloning to construct the three vectors with the appropriate epitope fusions at the amino termini of the candidate proteins for our analyses (Fig. 1). This Gateway strategy expedited our studies because of the high efficiency of this system and because only entry clones needed to be confirmed by sequencing.

### Testing TRPML1 Interactors by Immunoprecipitation and Western Analysis

**Figure 2:**
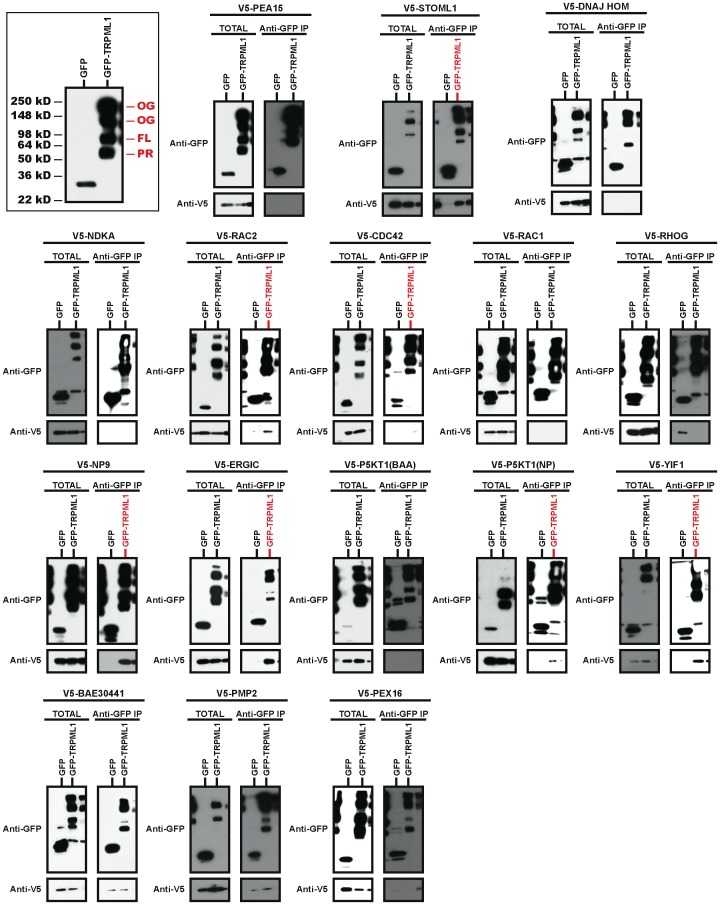
Immunoprecipitation Tests of Candidate Interactors. Plasmids expressing GFP (control) or murine GFP-TRPML1 protein were co-transfected with plasmids expressing V5 fusions to candidate interactors into HeLa cells. Anti-GFP immunoprecipitation was performed on lysates. Left panels are Western blots that show total expression in lysates and right panels are Western blots of immunoprecipitates (IP). Red lettering indicates lanes exhibiting co-immunoprecipitation with GFP-TRPML1. The top left, boxed panel shows a typical pattern of GFP-TRPML1 bands: PR  =  processed/cleaved; FL  =  full-length; OG  =  oligomer.

We transfected HeLa cells (because of their high transfection efficiency) with plasmids that express GFP (negative control) or GFP-TRPML1, along with plasmids that express V5 epitope-fusions of candidate interactors. We then immunoprecipitated GFP-TRPML1 using a bead-conjugated anti-GFP antibody in the absence of Ca^2+^ and assessed by Western blot the co-immunoprecipitation of V5-fused putative partner proteins. The Western blots of GFP-TRPML1 total lysate and immunoprecipitation samples probed with anti-GFP antibody typically contained several bands that correspond to an ∼55 kD cleaved GFP-TRPML1 (PR in Fig. 2), ∼93 kD full-length GFP-TRPML1 (FL in Fig. 2), and two higher molecular weight bands that are presumed to be GFP-TRPML1 oligomers (OG in Fig. 2); these bands are consistent with previous studies overexpressing TRPML1 [9], [29], [38].

Of the potential interactors identified by Immunoprecipitation/Mass Spectrometry, we confirmed that V5-STOML1 and V5-NP9 co-immunoprecipitated very strongly with GFP-TRPML1; V5-Rac2 co-immunoprecipitated strongly with GFP-TRPML1, and V5-Cdc42 co-immunoprecipitated weakly but reproducibly with GFP-TRPML1 (Fig. 2; Table 1). Rac1 and RhoG, the Rac2 and Cdc42 homologues that were identified by Immunoprecipitation/Mass Spectrometry, did not co-immunoprecipitate with TRPML1 (Fig. 2; Table 1). Thus, four of the seven candidate interactors re-tested positive with TRPML1 in this secondary screen; the other three proteins, PEA-15, DNAJ HOM, and NDKA represent false-positive interactors from the Immunoprecipitation/Mass Spectrometry screen (Fig. 2; Table 1).

**Table 1:** TRPML1 Interactions Summary.

Protein	IP (−Ca^2+^)		Split-Ub YTH		Co-localization		
PEA-15		−		+/−		+	
STOML1		++*		−		++	
DNAJ HOM		−		−		−	
NDKA		−		++		−	
Rac2		+		++		++	
Cdc42		+/−		++		++	
Rac1		−		+		−	
RhoG		−		−		++	
NP9		++		+		++	
ERGIC		++		+		−	
P5KT1 (BAA)		−		ND		−	
P5KT1 (NP)		+		−		−	
YIF1		++		+		−	
BAE		−		+		+	
PMP2		−		++		−	
PEX16		−		++		−	

Qualitative assessment of interactions. Plus signs indicate interaction; minus signs indicate lack of interaction. Immunoprecipitation interaction strength was based on length of time before anti-V5 band appeared (anti-GFP bands appeared with 1 second of film exposure). Asterisk indicates that endogenous mouse STOML1 co-immunoprecipitates with GFP-TRPML1 in murine RAW264.7 macrophages. Split-Ubiquitin Yeast Two-Hybrid interaction strength was based on amount of growth on plates. Co-localization interaction strength is scored as one plus sign for every 25% co-localization (average). Dotted line separates candidate interactors identified by co-IP (above) or by Split-Ub YTH (below).

Of the candidate interactors identified by SU-YTH, we found that V5-ERGIC and V5-YIF1 co-immunoprecipitated very strongly with GFP-TRPML1, and V5-P5KT1(NP_032872) co-immunoprecipitated strongly with GFP-TRPML1 (Fig. 2; Table 1). P5KT1(BAA13031), which was originally identified as a TRPML1 interactor by the SU-YTH screen, did not co-immunoprecipitate with TRPML1 (Fig. 2; Table 1). Together, these observations indicate that neither the Immunoprecipitate/Mass Spectrometry not the SU-YTH approach was saturating for the identification of the TRPML1 interactome.

### Testing Endogenous TRPML1 Interactions by Immunoprecipitation and Western Analysis

**Figure 3:**
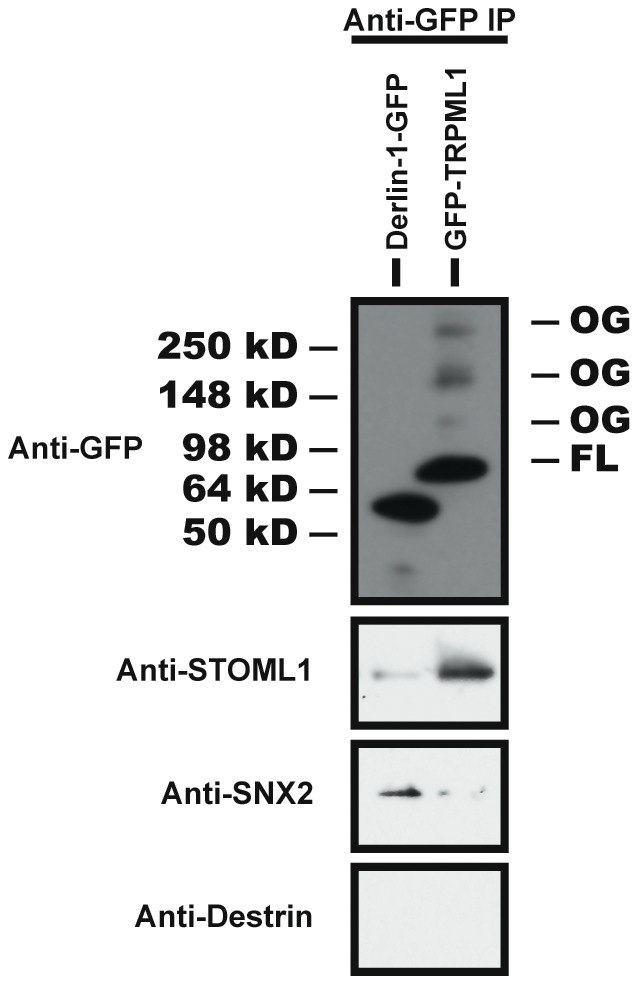
Immunoprecipitation Tests of Endogenous Proteins. Lysis and anti-GFP immunoprecipitation was performed on murine RAW264.7 macrophages stably expressing mouse GFP-TRPML1 or Derlin-1-GFP. Sorting Nexin 2 (SNX2) is a protein previously shown to interact with Derlin-1; Destrin was used as a negative control. FL  =  full-length; OG  =  oligomer.

To confirm the validity of our immunoprecipitation assay using overexpressed GFP-TRPML1 and V5-fused candidate proteins in HeLa cells, we assayed whether endogenous mouse proteins co-immunoprecipitate with mouse GFP-TRPML1 in the absence of Ca^2+^. We immunoprecipitated GFP-TRPML1 or Derlin-1-GFP from stable RAW264.7 clones that express these fusion proteins; in these clones GFP-TRPML1 localizes predominantly to late endosomes/lysosomes and Derlin-1-GFP localizes predominantly to the endoplasmic reticulum with some Derlin-1 GFP localizing in endosomes [19], [34]. The full-length (93 kD) GFP-TRPML1 is the main form of this protein in RAW264.7 cells, although we could also detect some higher molecular weight isoforms (Fig. 3). Derlin-1-GFP migrated at the predicted size of 55 kD by SDS-PAGE.

We assayed commercial antibodies that had been raised against STOML1, Rac2, and ERGIC; only the mouse anti-STOML1 antibody recognized a protein of the predicted size in RAW264.7 lysates. We therefore confined our analysis to STOML1. STOML1 co-immunoprecipitated preferentially with GFP-TRPML1 (Fig. 3). In contrast, Sorting Nexin 2 (SNX2) co-immunoprecipitated preferentially with Derlin-1-GFP, consistent with our previous study showing that SNX2 associates with Derlin-1 (Fig. 3) [31]. The negative control Destrin, an actin-binding protein, did not co-immunoprecipitate with either GFP-TRPML1 or Derlin-1-GFP (Fig. 3) [39]. Thus endogenous STOML1 interacts with TRPML1 (Table 1).

### Testing TRPML1 Interactors by SU-YTH

**Figure 4:**
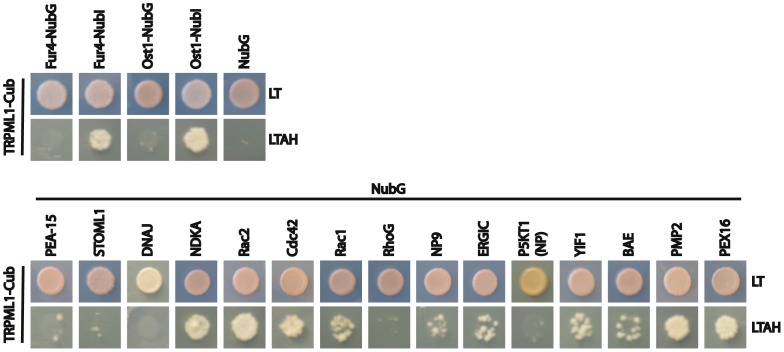
Split-Ubiquitin Yeast Two-Hybrid Tests of Candidate Interactors. The same number of cells of yeast strains carrying indicated constructs were spotted on SD–leu –trp (LT) plates that select for plasmids or SD–leu –trp –ade – his +1 mM 3-AT (LTAH) plates that assay for interaction. Fur4-NubG, Ost1-NubG, and NubG are negative controls; Fur4-NubI and Ost1-NubI are positive controls.

The limited overlap in potential interactors using our two approaches was initially surprising, but not completely inexplicable given the potential challenges of the SU-YTH screen. Although this approach allows full-length proteins to be assayed, the disadvantage of SU-YTH is that it represents an artificial system where proteins are assayed in *S. cerevisiae* where they are not normally expressed and perhaps at levels and locations that are non-native. For example, based on interactions with Fur4-NubI (plasma membrane) and Ost1-NubI (endoplasmic reticulum), TRPML1-Cub-LexA-VP16 predominantly localizes to the endoplasmic reticulum (Fig. 4). Thus, proteins that associate with TRPML1 but that do not localize to the endoplasmic reticulum may not be captured in this assay (false-negatives). Finally, some exogenous proteins, such as full-length P5KT1(BAA13031), are toxic to *S. cerevisiae* and cannot be studied using the SU-YTH approach.

Of the candidate interactors identified by SU-YTH screens, we confirmed that TRPML1 still associated with their full-length versions by SU-YTH, with the exception of P5KT1(NP_032872) (Fig. 4; Table 1). While we do not know why P5KT1(NP_032872) failed to associate with TRPML1 by SU-YTH, we suspect that this protein is misfolded in *S. cerevisiae* since expression of the homologous P5KT1(BAA13031) protein is toxic to yeast.

We directly tested potential interactors isolated by the Immunoprecipitation/Mass Spectrometry approach in the SU-YTH screen. Through this analysis, we confirmed that TRPML1 associates with NDKA, Rac2, Cdc42, and NP9 by SU-YTH (Fig. 4; Table 1). TRPML1 also associated with Rac1 but not RhoG. Although all of these proteins were missed by the initial SU-YTH screen, their confirmation in this directed approach strongly suggests that they are strong candidate TRPML1 interactors. Several other proteins did not interact with TRPML1 by the SU-YTH assay. Of note, our failure to detect a STOML1-TRPML1 SU-YTH interaction is likely due to NubG-STOML1 localizing to vacuoles of yeast where there is little TRPML1-Cub-LexA-VP16 (Fig. 4).

### Testing Co-localization with TRPML1

As another approach to validate candidate TRPML1 interactors, we assayed co-localization of the identified proteins with GFP-TRPML1 in RAW264.7 cells. GFP-TRPML1 predominantly localizes to late endosome and lysosomes of these murine macrophages at steady state, similar to GFP-TRPML1's localization in other cell types [19]. While we only analyzed the cells that expressed minimal levels of fusion proteins that were still detectable by microscopy, we cannot rule out that some co-localization with GFP-TRPML1 may be a consequence of the overexpression of the fusion proteins.

Some TagRFP(S158T)-fused candidate proteins were not detectable by microscopy either because the steady state levels of these proteins were too low in the transfected cells and/or because the TagRFP(S158T) epitope affects the folding and hence stability of the fusion proteins. For these proteins, we used the V5-fused forms and performed immunofluorescence analysis on the cells to assay co-localization with GFP-TRPML1; the V5 epitope (GKPIPNPLLGLDST) is relatively small and is hence less likely to interfere with the folding of the fusion protein.

**Figure 5:**
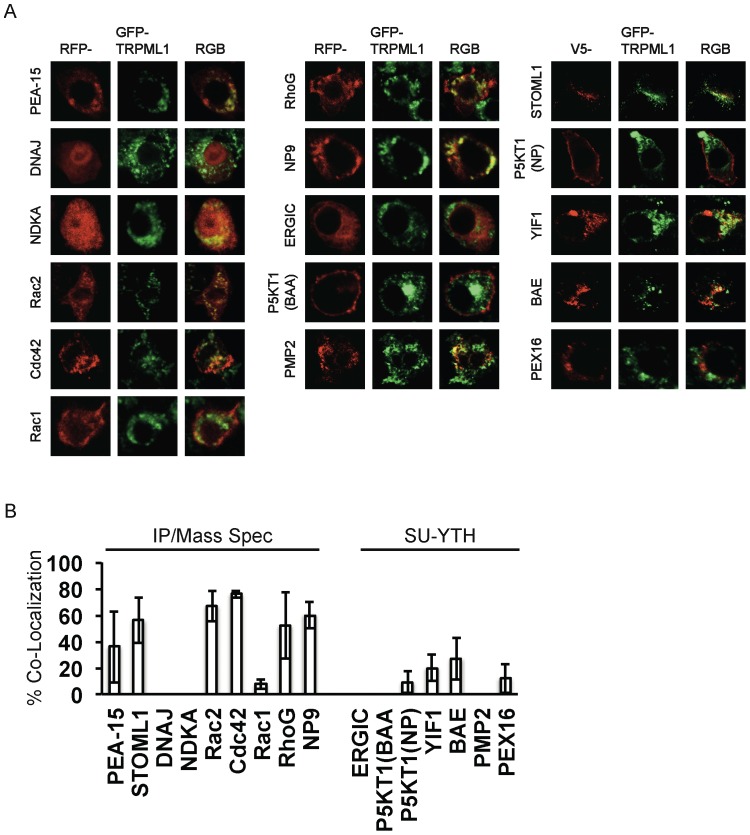
Co-Localization Tests of Candidate Interactors. *A*, Plasmids expressing TagRFP(S158T) or V5 fusions to candidate interactors were transfected into RAW264.7 macrophages that stably express GFP-TRPML1. Confocal microscopy was done on fixed cells. Cells transfected with V5-X proteins were immunostained to localize the V5 fusion proteins. *B*, Quantitation of percent of TagRFP(S15T)/V5-X discrete structures that also have GFP-TRPML1. Bars represent standard deviations.

Of the candidate interactors identified by the Immunoprecipitation/Mass Spectrometry screen, TRPML1 showed significant co-localization with PEA-15, STOML1, Rac2, Cdc42, RhoG (but not Rac1), and NP9 (Fig. 5; Table 1). Of the candidate interactors identified by the SU-YTH screen, TRPML1 showed a low level of co-localization with YIF1 and BAE30441 (Fig. 5; Table 1).

## Discussion

We describe two large-scale screens for TRPML1 interactors, the first based on Immunoprecipitation/Mass Spectrometry and the second using SU-YTH assays. Each of these screens identified a list of potential TRPML1 interactors with minimal overlap. The only protein identified by both screens was isoform 3 of glyceraldehyde 3-phosphate dehydrogenase, but the screens also identified homologous proteins for the alpha subunit of a sodium channel protein and for cadherin-like proteins.

To determine the validity of the Immunoprecipitation/Mass Spectrometry and the SU-YTH screens, we carried out an unbiased survey of some potential interactors identified by each screen. Of seven proteins tested from the Immunoprecipitation/Mass Spectrometry list, four proteins, Rac2, Cdc42, NP9, and STOML1, are strong candidate interactors of TRPML1, showing association with TRPML1 using both Immunoprecipitation/Western, either at endogenous or elevated levels, and SU-YTH assays (Table 1). Of six proteins tested from the SU-YTH list, two proteins, ERGIC and YIF1, are strong candidate interactors of TRPML1, showing association with TRPML1 using both Immunoprecipitation/Western and SU-YTH assays (Table 1). P5KT1(NP_032872) is also a candidate interactor because it associated with TRPML1 using Immunoprecipitation/Western but not SU-YTH assays (Table 1). Thus, while it is clear that, as expected, both the Immunoprecipitation/Mass Spectrometry and the SU-YTH screens yielded false-positive results, both screens also missed candidate interactors. This observation suggests that a more comprehensive identification of interactors of a protein of interest requires multiple screens. Furthermore, given that interacting proteins could be missed by each individual screen, further analyses should not be confined solely to the proteins that are preliminarily identified by both screens. As such, the potential TRPML1 interactors we identified in our screens are a good resource for identifying proteins that interact with TRPML1 (Tables S2, S3).

The molecular identities of the candidate TRPML1 interactors suggest potential roles in TRPML1 biology. In other systems, both ERGIC and Golgi 3 (ERGIC) and Yip1 Interacting Factor (YIF1) have been implicated in ER/Golgi transport, suggesting that these proteins may mediate the biosynthetic transport of TRPML1 protein [40], [41].

STOML1 is an integral membrane protein that had previously been shown to localize to late endosomes/lysosomes [42]. Intriguingly, STOML1 has a lumenal sterol carrier protein-2 (SCP-2) domain. This observation suggests that TRPML1 may function in lipid transport from late endosomes/lysosomes through its interactions with STOML1, which is consistent with a reduced efficiency of this lipid transport step in MLIV cells.

Rac2 and Cdc42 are small GTPases that regulate the actin cytoskeleton [43]. Rac2 and Cdc42 may be involved in the lysosome biogenesis (also referred to as lysosome reformation) and/or lysosome exocytosis functions of TRPML1,because Rac2 and Cdc42 localize to both late endosomes and lysosomes with TRPML1 and also to the plasma membrane (Fig. 5) [23], [44], [45]. We had previously showed that CUP-5, the *Caenorhabditis elegans* orthologue of TRPML1 functions in lysosome biogenesis [45]. Subsequently, the *C. elegans* small GTPase RAB-2 was also shown to function in lysosome biogenesis in the same cells as CUP-5 [46], [47]. Thus *C. elegans* RAB-2 may be the worm homologue of mammalian Rac2 mediating the lysosomal transport functions of CUP-5.

Phosphatidylinositol 4-Phosphate 5-Kinase Type I-Beta (P5KT1) generates phospholipid PI(4,5)P2, which functions as a modulator of several membrane transport and signaling processes and as a regulator of the actin cytoskeleton [48], [49], [50]. P5KT1 may function with TRPML1 during lysosome exocytosis given the strong localization of P5KT1 to the plasma membrane (Fig. 5). Supporting this potential lysosome exocytosis function, regulation of PI(4,5)P2 at the plasma membrane is critical during exocytosis, including of lysosome-related organelles [51], [52], [53].

The novel protein Likely Orthologue of Human FAM11A Family with Sequence Similarity 11, Member (NP9) is a multi-spanning integral membrane protein of unknown function. NP9 co-localizes with TRPML1 on late endosomes/lysosomes, suggesting possible roles in one of TRPML1's trafficking and/or channel functions in these compartments.

It may be significant that some of the candidate TRPML1 interactors possibly align with functions of TRPML1 that were proposed based on observed defects in MLIV cells or in models of MLIV. It is possible that TRPML1 has one primary function, for example lysosome biogenesis in most cells; in the absence of TRPML1, lysosome biogenesis is inefficient leading to defective lysosomes and thus indirectly to other defects like lipid transport to Golgi apparatus and lysosome exocytosis. However, our candidate interactors suggest the alternative explanation that TRPML1 directly functions in all of these processes through association with distinct complexes of proteins. Future analyses will test this prediction and elucidate the significance of these interactions.

## Supporting Information

Table S1 **Plasmids used in this study.**
(DOCX)

Table S2 **List of proteins identified by Immunoprecipitation/MassSpectrometry.**
(DOCX)

Table S3 **List of proteins identified by Split Ubiquitin Yeast Two-HybridScreens.**
(DOCX)
