# Supplementary material for: Systematic Screens for Proteins That Interact with the Mucolipidosis Type IV Protein TRPML1
Source: PLoS One. 2013 Feb 13;8(2):e56780. doi: 10.1371/journal.pone.0056780 (PMC3572064; doi:10.1371/journal.pone.0056780)
Supplement: Table S1 — Plasmids used in this study. (DOCX) [file pone.0056780.s001.docx]

| **Supplemental Table S1. Candidate Interactors Plasmids** | | | | |
| --- | --- | --- | --- | --- |
| Gene/Protein | Plasmid | Epitope | Epitope fused to | Vector backbone |
| PEA-15 | pHD527 | V5 | NH_2_-terminus | pcDNA3.1/nV5-DEST |
| PEA-15 | pHD465 | NubG | NH_2_-terminus | pPR3-N-GTWY |
| PEA-5 | pHD466 | TagRFP(S158T) | NH_2_-terminus | pDEST-N-TagRFP |
| STOML1 | pHD580 | V5 | NH_2_-terminus | pcDNA3.1/nV5-DEST |
| STOML1 | pHD578 | NubG | NH_2_-terminus | pPR3-N-GTWY |
| STOML1 | pHD579 | TagRFP(S158T) | NH_2_-terminus | pDEST-N-TagRFP |
| DNAJ | pHD603 | V5 | NH_2_-terminus | pcDNA3.1/nV5-DEST |
| DNAJ | pHD570 | NubG | NH_2_-terminus | pPR3-N-GTWY |
| DNAJ | pHD589 | TagRFP(S158T) | NH_2_-terminus | pDEST-N-TagRFP |
| NDKA | pHD569 | V5 | NH_2_-terminus | pcDNA3.1/nV5-DEST |
| NDKA | pHD567 | NubG | NH_2_-terminus | pPR3-N-GTWY |
| NDKA | pHD568 | TagRFP(S158T) | NH_2_-terminus | pDEST-N-TagRFP |
| Rac2 | pHD528 | V5 | NH_2_-terminus | pcDNA3.1/nV5-DEST |
| Rac2 | pHD467 | NubG | NH_2_-terminus | pPR3-N-GTWY |
| Rac2 | pHD468 | TagRFP(S158T) | NH_2_-terminus | pDEST-N-TagRFP |
| Cdc42 | pHD608 | V5 | NH_2_-terminus | pcDNA3.1/nV5-DEST |
| Cdc42 | pHD613 | NubG | NH_2_-terminus | pPR3-N-GTWY |
| Cdc42 | pHD788 | TagRFP(S158T) | NH_2_-terminus | pDEST-N-TagRFP |
| Rac1 | pHD606 | V5 | NH_2_-terminus | pcDNA3.1/nV5-DEST |
| Rac1 | pHD611 | NubG | NH_2_-terminus | pPR3-N-GTWY |
| Rac1 | pHD781 | TagRFP(S158T) | NH_2_-terminus | pDEST-N-TagRFP |
| RhoG | pHD607 | V5 | NH_2_-terminus | pcDNA3.1/nV5-DEST |
| RhoG | pHD612 | NubG | NH_2_-terminus | pPR3-N-GTWY |
| RhoG | pHD787 | TagRFP(S158T) | NH_2_-terminus | pDEST-N-TagRFP |
| NP9 | pHD534 | V5 | NH_2_-terminus | pcDNA3.1/nV5-DEST |
| NP9 | pHD478 | NubG | NH_2_-terminus | pPR3-N-GTWY |
| NP9 | pHD479 | TagRFP(S158T) | NH_2_-terminus | pDEST-N-TagRFP |
| ERGIC | pHD532 | V5 | NH_2_-terminus | pcDNA3.1/nV5-DEST |
| ERGIC | pHD482 | NubG | NH_2_-terminus | pPR3-N-GTWY |
| ERGIC | pHD475 | TagRFP(S158T) | NH_2_-terminus | pDEST-N-TagRFP |
| P5KT1 (BAA13031) | pHD795 | V5 | NH_2_-terminus | pcDNA3.1/nV5-DEST |
| P5KT1 (BAA13031) | pHD676 | NubG | NH_2_-terminus | pPR3-N-GTWY |
| P5KT1 (BAA13031) | pHD789 | TagRFP(S158T) | NH_2_-terminus | pDEST-N-TagRFP |
| P5KT1 (NP_032872) | pHD738 | V5 | NH_2_-terminus | pcDNA3.1/nV5-DEST |
| P5KT1 (NP_032872) | pHD706 | NubG | NH_2_-terminus | pPR3-N-GTWY |
| P5KT1 (NP_032872) | pHD791 | TagRFP(S158T) | NH_2_-terminus | pDEST-N-TagRFP |
| YIF1 | pHD576 | V5 | NH_2_-terminus | pcDNA3.1/nV5-DEST |
| YIF1 | pHD574 | NubG | NH_2_-terminus | pPR3-N-GTWY |
| YIF1 | pHD575 | TagRFP(S158T) | NH_2_-terminus | pDEST-N-TagRFP |
| BAE | pHD577 | V5 | NH_2_-terminus | pcDNA3.1/nV5-DEST |
| BAE | pHD591 | NubG | NH_2_-terminus | pPR3-N-GTWY |
| BAE | pHD582 | TagRFP(S158T) | NH_2_-terminus | pDEST-N-TagRFP |
| PMP2 | pHD587 | V5 | NH_2_-terminus | pcDNA3.1/nV5-DEST |
| PMP2 | pHD626 | NubG | NH_2_-terminus | pPR3-N-GTWY |
| PMP2 | pHD779 | TagRFP(S158T) | NH_2_-terminus | pDEST-N-TagRFP |
| PEX16 | pHD604 | V5 | NH_2_-terminus | pcDNA3.1/nV5-DEST |
| PEX16 | pHD628 | NubG | NH_2_-terminus | pPR3-N-GTWY |
| PEX16 | pHD780 | TagRFP(S158T) | NH_2_-terminus | pDEST-N-TagRFP |
