# Supplementary material for: Systematic Screens for Proteins That Interact with the Mucolipidosis Type IV Protein TRPML1
Source: PLoS One. 2013 Feb 13;8(2):e56780. doi: 10.1371/journal.pone.0056780 (PMC3572064; doi:10.1371/journal.pone.0056780)
Supplement: Table S2 — List of proteins identified by Immunoprecipitation/MassSpectrometry. (DOCX) [file pone.0056780.s002.docx]

| \| \| **Supplemental Table S2. Immunoprecipitation/Mass Spectrometry Data** \| \| --- \| \| \| --- \| --- \| | | | | |
| --- | --- | --- | --- | --- | --- | --- |
| Protein | NCBI Number | Unique Peptides | *P* peptide (%) | *P* Protein (%) |

| Isoform 1 of Astrocytic phosphoprotein PEA-15 | Q62048-1 | 1 | 95 | 92 |
| --- | --- | --- | --- | --- |

| Stomatin-like protein 1 (STOML1) | Q8C I66 | 2 | 95 | 99 |
| --- | --- | --- | --- | --- |

| DnaJ homolog subfamily B member 1 (DNAJ) | Q9QYJ3 | 1 | 95 | 92 |
| --- | --- | --- | --- | --- |
| Nucleoside diphosphate kinase A (NDKA) | P15532 | 2 | 95 | 99 |
| Rac 2 | Q05144 | 1 | 95 | 92 |
| Isoform 2 of CDC42 (Cdc42) | P60766-2 | 1 | 95 | 92 |
| novel protein (likely orthologe of human FAM11A family with sequence similarity 11, member A) (NP9) | CAM23469 | 1 | 95 | 92 |
| Protocadherin-19 precursor | Q80TF3 | 1 | 95 | 92 |
| Protocadherin 1 | NP_083633.2 | 1 | 95 | 92 |
| Cadherin 9 | P70407 | 1 | 93 | 90 |
| Similar to Glyceraldehyde-3-phosphate dehydrogenase isoform 3 | [XP_001479421.2](http://www.ncbi.nlm.nih.gov/protein/309266468?report=genbank&log$=prottop&blast_rank=1&RID=AJY4RHM5013) | 3 | 95 | 100 |
| Isoform 1 of Sodium channel protein type 10 subunit alpha | Q6QIY3-1 | 1 | 95 | 92 |

| 60S ribosomal protein L14 | Q9CR57 | 1 | 95 | 92 |
| --- | --- | --- | --- | --- |

| Histone H2B type 1-A | P70696 | 1 | 95 | 97 |
| --- | --- | --- | --- | --- |
| Tubulin alpha-1 chain | P68369 | 6 | 95 | 100 |

| ADAM 1b precursor | Q8R534 | 1 | 95 | 92 |
| --- | --- | --- | --- | --- |
| Adult male spinal cord cDNA, RIKEN full-length enriched library, clone:A330099J10 product:similar to Serologically defined breast cancer antigen NY-BR-15-like protein (Fragment) | NP_001191912.1 | 1 | 95 | 92 |
| Cathepsin B precursor | P10605 | 3 | 95 | 100 |
| Isoform 1 of Mucolipin-1 | Q99J21-1 | 2 | 95 | 100 |
| 2156 kDa protein | A2ASS6-1 | 1 | 90 | 99 |
| 26S proteasome non-ATPase regulatory subunit 1 | Q3TXS7 | 1 | 95 | 96 |
| Desmoglein-2 precursor | O55111 | 2 | 95 | 99 |
| Prohibitin | P67778 | 2 | 95 | 99 |
| Zinc finger, CCHC domain containing 2 isoform 1 | Q69ZB8-2 | 2 | 93 | 99 |
| Fibrillin-1 precursor | Q61554 | 1 | 94 | 98 |
| Agrin | A2ASQ1-2 | 1 | 95 | 98 |
| Similar to Myosin light polypeptide 6 | XP_998567.3 | 1 | 95 | 97 |
| Retinoic acid receptor gamma-B | P18911-2 | 1 | 95 | 96 |
| 10 days neonate cerebellum cDNA, RIKEN full-length enriched library, clone:B930053E03:weakly similar to G2 PROTEIN | NP_001028519..2 | 1 | 95 | 96 |
| IsIr2 protein | Q5RKR3 | 1 | 95 | 96 |
| T-complex protein 1 subunit zeta | P80317 | 1 | 95 | 92 |
| Adult male testis cDNA, RIKEN full-length enriched library, clone:4930507C10 product:similar to CDNA FLJ13840 FIS, CLONE THYRO1000783, MODERATELY SIMILAR TO XENOPUS LAEVIS TAIL-SPECIFIC THYROID HORMONE UP-REGULATED (GENE5) MRNA | Q9D2G9-2 | 1 | 95 | 92 |
| Prolyl 3-hydroxylase 2 precursor | Q8CG71 | 1 | 95 | 92 |
| Arid3b protein | Q9Z1N7-2 | 1 | 95 | 92 |
| Solute carrier family 35, member F5 | Q8R314 | 1 | 95 | 92 |
| F-actin capping protein alpha-1 subunit | P47753 | 1 | 95 | 92 |
| Microcephalin | Q7TT79 | 1 | 95 | 92 |
| Stretch-responsive fibronectin protein type 3 | Q70KF4 | 1 | 95 | 92 |
| 24 kDa protein | Q3URD3-4 | 1 | 95 | 92 |
| Gene model 996 | A2AJA9-1 | 1 | 94 | 91 |
| Kelch-like protein 10 | Q9D5V2 | 1 | 95 | 92 |
| Tubulin beta-2B chain | Q9CWF2 | 1 | 95 | 92 |
| Regulator of G-protein signaling 19 | Q9CX84 | 1 | 95 | 92 |
| Alpha-protein kinase 1 | Q9CXB8 | 1 | 95 | 92 |
| Adult male small intestine cDNA, RIKEN full-length enriched library, clone:2010109A12 product:hypothetical Microbodies C-terminal targeting signal containing protein, full insert sequence | XP_001475763.1 | 1 | 95 | 92 |
| Isoform 1 of Protein phosphatase 1 regulatory subunit 14D | Q7TT52-1 | 1 | 95 | 92 |
| Immunoresponsive gene 1 | P54987 | 1 | 95 | 92 |
| 60S ribosomal protein L4 | Q9D8E6 | 1 | 95 | 92 |
| Nicolin-1 | Q9CQM0 | 1 | 95 | 92 |
| Hypothetical protein LOC76768 | NP_001074551.1 | 1 | 95 | 92 |
| ERBB receptor feedback inhibitor 1 | Q99JZ7 | 1 | 95 | 92 |
| Hematopoietic lineage cell-specific protein | P49710 | 1 | 95 | 92 |
| Adult male testis cDNA, RIKEN full-length enriched library, clone:1700011N24 product: hypothetical Retroviral-type aspartic protease containing protein, full insert sequence | Q9DAF3 | 1 | 95 | 92 |
| ATP synthase O subunit, mitochondrial precursor | Q9DB20 | 1 | 95 | 92 |
| Isoform 1 of Estradiol 17-beta-dehydrogenase 12 | Q70503-1 | 1 | 95 | 92 |
| Isoform 1 of Adenomatous polyposis coli protein | Q61315-1 | 1 | 95 | 92 |
| Coatomer subunit beta | Q9JIF7 | 1 | 95 | 92 |
| Isoform 1 of Translocon-associated protein subunit gamma | Q9DCF9-1 | 1 | 95 | 92 |
| Nuclease sensitive element-binding protein 1 | P62960 | 1 | 95 | 92 |
| NADH dehydrogenase [ubiquinone] 1 alpha subcomplex subunit 8 | Q9DCJ5 | 1 | 95 | 92 |
| Isoform PI-VDAC1 of Voltage-dependent anion-selective channel protein 1 | Q60932-1 | 1 | 95 | 92 |
| 40S ribosomal protein S27-like protein | Q6ZWY3 | 1 | 95 | 92 |
| Ubiquitin-conjugating enzyme E2 D2 | P62838 | 1 | 95 | 92 |
| Ephrin type-A receptor 6 precursor | BAE43358.1 | 1 | 95 | 92 |
| Olfactory receptor MOR277-1 | NP_667088.1 | 1 | 95 | 92 |
| Probable ATP-dependent RNA helicase DDX41 | Q91VN6 | 1 | 95 | 92 |
| Olfactory receptor MOR135-3 | NP_667234.1 | 1 | 95 | 92 |
| 5-hydroxytryptamine 6 receptor | Q9R1C8 | 1 | 95 | 92 |
| Proteasome subunit beta type 2 | Q9R1P3 | 1 | 95 | 92 |
| Ornithine aminotransferase, mitochondrial precursor | P29758 | 1 | 95 | 92 |
| 8 days embryo whole body cDNA, RIKEN full-length enriched library, clone:5730457G12 product: small inducible cytokine subfamily E, member 1, full insert sequence | P31230 | 1 | 95 | 92 |
| Integrin beta-1 precursor | P09055 | 1 | 95 | 92 |
| Isoform 1 of Uncharacterized aarF domain-containing protein kinase 1 precursor | Q9D0L4-1 | 1 | 95 | 92 |
| Coiled-coil-helix-coiled-coil-helix domain-containing protein 5 | Q9CQP3 | 1 | 95 | 92 |
| Matrix metalloproteinase-14 precursor | P53690 | 1 | 95 | 92 |
| Nuclear protein Hcc-1 | Q9D1J3 | 1 | 95 | 92 |
| Adult male spinal cord cDNA, RIKEN full-length enriched library, clone:A330085J21 product:hypothetical protein, full insert sequence | NP_083683.1 | 1 | 95 | 92 |
| Ataxin-3 | Q9CVD2 | 1 | 95 | 92 |
| Splicing factor 3b, subunit 4 | Q8QZY9 | 1 | 95 | 92 |
| Isoform 1 of Zinc finger protein GLIS1 | Q8K1M4-1 | 1 | 95 | 92 |
| Isoform 2 of Protein KIAA1958 homolog | Q8C4P0-2 | 1 | 95 | 92 |
| Isoform 1 of HIV Tat-specific factor 1 homolog | Q8BGC0-1 | 1 | 95 | 92 |
| 12 days embryo male wolffian duct includes surrounding region cDNA, RIKEN full-length enriched library, clone: 6720467K22 product:similar to VACUOLAR ATP SYNTHASE SUBUNIT G 3 | Q8BMC1 | 1 | 95 | 92 |
| Uricase | P25688 | 1 | 95 | 92 |
| Adult male testis cDNA, RIKEN full-length enriched library, clone: 4932435K23 product:SERUM INHIBITED-RELATED PROTEIN homolog | A2RSY1-2 | 1 | 95 | 92 |
| CDNA sequence BC057170 | NP_766365.1 | 1 | 95 | 92 |
| 0 day neonate cerebellum cDNA, RIKEN full-length enriched library, clone:C230001I03 product:hypothetical Arginine-rich region profile containing protein, full insert sequence | BAE37524.1 | 1 | 95 | 92 |
| Alpha isoform of regulatory subunit B”, protein phosphatase 2 isoform 5 | BAC37349.1 | 1 | 95 | 92 |
| Adult male testis cDNA, RIKEN full-length enriched library, clone:4921523A10 product:hypothetical Protein phosphatase 2C domain containing protein, full insert sequence | Q8BVT6 | 1 | 95 | 92 |
| Beta-galactoside alpha-1,2-fucosyltransferase | O09160 | 1 | 95 | 92 |
| Trinucleotide repeat containing 6b isoform 1 | Q8BKI2-1 | 1 | 95 | 92 |
| Serine protease inhibitor A3B precursor | Q8BYY9 | 1 | 95 | 92 |
| Pogo transposable element with ZNF domain | Q8BZH4 | 1 | 95 | 92 |
| 10 days neonate cerebellum cDNA, RIKEN full-length enriched library, clone:6530425B06 product:hypothetical protein, full insert sequence | BAC27988.1 | 1 | 95 | 92 |
| NACHT, LRR and PYD-containing protein 10 | Q8CCN1 | 1 | 95 | 92 |
| Solute carrier family 1 (Neutral amino acid transporter), member 5 | AAH29873.1 | 1 | 95 | 92 |
| Neuron navigator 1 | Q8CH77-1 | 1 | 95 | 92 |
| 14-3-3 protein gamma | P61982 | 1 | 95 | 92 |
| Myosin light polypeptide 6B | Q8CI43 | 1 | 95 | 92 |
| Inositol 1,4,5-triphosphate 3-kinase B isoform 1 | NP_001074644.1 | 1 | 95 | 92 |
| REST compressor 1 | Q8CFE3 | 1 | 95 | 92 |
| DEAH | NP_848467.1 | 1 | 95 | 92 |
| Trafficking protein particle complex subunit 1 | Q5NCF2 | 1 | 95 | 92 |
| Isoform 1 of Ubiquitin carboxyl-terminal hydrolase 28 | Q5I043-1 | 1 | 95 | 92 |
| 12 days embryo embryonic body between diaphragm region and neck cDNA, RIKEN full-length enriched library, clone: 9430001H05 product:PAXNEB PROTEIN homolog | Q9ER73 | 1 | 95 | 92 |
| 12 days embryo spinal cord cDNA, RIKEN full-length enriched library, clone:C530044P16 product:cholinergic receptor, nicotinic, beta polypeptide 2 | NP_033732.2 | 1 | 95 | 92 |
| Transcriptional regulator Kaiso | Q8BN78 | 1 | 95 | 92 |
| 7 days embryo whole body cDNA, RIKEN full-length enriched library, clone:C430003J15 product: hypothetical Nucleic acid-binding proteins structure containing protein, full insert sequence | Q8BGW5-1 | 1 | 95 | 92 |
| Bifunctional 3’-phosphoadenosine 5’-phosphosulfate synthetase 1 | Q60967 | 1 | 95 | 92 |
| Cadherin EGF LAG seven-pass G-type receptor 2 precursor | Q9R0M0-1 | 1 | 95 | 92 |
| 17 days embryo kidney cDNA, RIKEN full-length enriched library, clone:I920078E17 product:CDC-like kinase 3, full insert sequence | O35492 | 1 | 95 | 92 |
| 8 days embryo whole body cDNA, RIKEN full-length enriched library, clone:5730439H01 product:UMP-CMP KINASE homolog | Q9DBP5 | 1 | 95 | 92 |
| Isoform 1 of Epsin-2 | Q8CHU3-1 | 1 | 95 | 92 |
| Mrpl55 protein | Q9CZ83-2 | 1 | 95 | 92 |
| Similar to Potassium voltage-gated channel subfamily G member 2 | NP_001177302.1 | 1 | 95 | 92 |
| Rosbin, round spermatid basic protein 1 | Q80T69 | 1 | 95 | 92 |
| 10 days neonate skin cDNA, RIKEN full-length enriched library, clone: 4732485E21 product:weakly similar to Zinc finger protein 408 | NP_001028623.1 | 1 | 95 | 92 |
| Coiled-coil domain-containing protein 27 | Q3V036 | 1 | 95 | 92 |
| Serpinb3d | NP_958764.1 | 1 | 95 | 92 |
| Hemogen | Q9ERZ0 | 1 | 95 | 92 |
| Anti-myosin immunoglobulin heavy chain variable region (Fragment) | AAF69325.1 | 1 | 95 | 92 |
| Anti-myosin immunoglobulin heavy chain variable region (Fragment) | AAF69321.1 | 1 | 95 | 92 |
| Hypothetical protein LOC433762 | AAH66867.1 | 1 | 95 | 92 |
| UDP-N-acetyl-alpha-D-galactosamine:polypeptide N-acetylgalactosaminyltransferase-like 1 isoform 1 | Q9JJ61 | 1 | 95 | 92 |
| Isoform 1 of Transmembrane emp24 domain-containing protein 10 precursor | Q9D1D4-1 | 1 | 95 | 92 |
| Stabilin-2 precursor | Q8R4U0 | 1 | 95 | 92 |
| Dachsous 1 | NP_001156415.1 | 1 | 95 | 92 |
| RIKEN cDNA 6230416J20 gene | NP_775576.2 | 1 | 95 | 92 |
| Slc7a5 protein | AAH13739.2 | 1 | 95 | 92 |
| 12 days embryo spinal ganglion cDNA, RIKEN full-length enriched library, clone:D130067D20 product:hypothetical Peptidase M1, membrane alanine aminopeptidase/ARM repeat fold containing protein, full insert sequence | Q8BXQ6-1 | 1 | 95 | 92 |
| Thyroid hormone receptor-associated protein 3 | Q569Z6 | 1 | 95 | 92 |
| Oxysterol-binding protein-like protein 7 isoform 3 | CAM24020.1 | 1 | 95 | 92 |
| Adult male testis cDNA, RIKEN full-length enriched library, clone:4931417M15 product:hypothetical Leucine-rich repeat/Leucine-rich repeat, typical subtype containing protein, full insert sequence | CAM19174.1 | 1 | 95 | 92 |
| Adult male diencephalons cDNA, RIKEN full-length enriched library, clone:9330101M15 product:hypothetical protein, full insert sequence | XP_003086271.1 | 1 | 95 | 92 |
| Activated spleen cDNA, RIKEN full-length enriched library, clone: F830006F03 product:hypothetical protein, full insert sequence | BAE33566.1 | 1 | 95 | 92 |
| Similar to melanoma antigen family A, 10 | BAC39706.1 | 1 | 95 | 92 |
| 42 kDa protein | P21708.5 | 1 | 95 | 92 |
| GPR158-like 1 receptor isoform 1 | BAC27053.1 | 1 | 95 | 92 |
| 109 kDa protein | NP_035554.2 | 1 | 95 | 92 |
| E3 ubiquitin-protein ligase LRSAM1 | Q80ZI6 | 1 | 95 | 92 |
| Isoform 1 of Centrosomal protein Cep290 | Q6A078-1 | 1 | 95 | 92 |
| Erythroblast cDNA, RIKEN full-length enriched library, clone:K0C0018C05 product:BA690P14.1 (Novel cyclin (Contains FLJ10895)) homolog | Q3TZI6-1 | 1 | 95 | 92 |
| CDNA sequence BC034076 | A2AJ76-1 | 1 | 95 | 92 |
| BMP-binding endothelial regulator protein precursor | Q8CJ69 | 1 | 94 | 92 |
| Probable ATP-dependent RNA helicase DDX6 | P54823 | 1 | 94 | 92 |
| Adult male tongue cDNA, RIKEN full-length enriched library, clone:2310012N15 product:T-complex expressed gene 1, full insert sequence | Q571F5-2 | 1 | 94 | 91 |
| Collagen alpha-2(IX) chain precursor | Q07643 | 1 | 94 | 91 |
| Isoform 5 of Discs large homolog 2 | Q91XM9-5 | 1 | 94 | 91 |
| 0 day neonate cerebellum cDNA, RIKEN full-length enriched library, clone:C230004C13 product:PROBABLE G PROTEIN-COUPLED RECEPTOR GPR21 homolog | Q8BX79 | 1 | 94 | 91 |
| Protein KIAA0286 | Q6ZQE4 | 1 | 94 | 91 |
| Probable palmitoyltransferase ZDHHC21 | Q9D270 | 1 | 94 | 90 |
| Nanos homolog 2 | P60322 | 1 | 93 | 90 |
| Schlafen 8 | NP_853523.2 | 1 | 93 | 90 |
| Similar to histocompatibility 2, Q region locus 1 | XP_894933.2 | 1 | 93 | 90 |
| Meprin 1 alpha | P28825 | 1 | 93 | 90 |
| Mitochondrial precursor proteins import receptor | Q9CZW5 | 1 | 93 | 90 |
| 78 kDa protein | P11021 | 1 | 95 | 92 |
| Isoform 1 of Ubiquitin carboxyl-terminal hydrolase 28 | [Q96RU2](http://www.uniprot.org/uniprot/Q96RU2) | 1 | 95 | 92 |
| Ia related protein | Q3JZA3 | 1 | 95 | 92 |
| Similar to CG14464-PA.3 | [XP_001121670.1](javascript:PopUpMenu2_Set(Menu_prot110758849);) | 1 | 95 | 92 |
| Similar to 10-formyltetrahydrofolate dehydrogenase (10-FTHFDH) (Aldehyde dehydrogenase 1 family member L1) isoform 2 | [NP_071992.1](javascript:PopUpMenu2_Set(Menu_prot11968144);) | 1 | 95 | 92 |
| Similar to Discs large homolog 5 | [NP_004738.3](javascript:PopUpMenu2_Set(Menu_prot95089461);) | 1 | 95 | 92 |
| Similar to cytoplasmic nuclear factor of activated T-cells 4 | [XP_690183.4](http://www.ncbi.nlm.nih.gov/protein/326665074) | 1 | 95 | 92 |
| Similar to testis-specific protein TSP-NY isoform a | Q6P9F0 | 1 | 95 | 92 |
| Hypothetical protein LOC75558 | [NP_083612.1](javascript:PopUpMenu2_Set(Menu_prot251823725);) | 1 | 94 | 92 |
| Similar to F-box and WD-40 domain protein FBXW14 | [XP_236641.4](javascript:PopUpMenu2_Set(Menu_prot109483971);) | 1 | 94 | 91 |
| This is the list of proteins that co-immunoprecipitated with GFP-TRPML1 but not with Derlin-1-GFP. *P* = probability, as determined by the Scaffold program. Rows highlighted in yellow are proteins that were retested in this study. Homologous proteins found by Mass Spectrometry (this table) and by Split-Ubiquitin Yeast Two-Hybrid (Supplementary Table 3) are highlighted with matching colors. | | | | |
