# Supplementary material for: Systematic Screens for Proteins That Interact with the Mucolipidosis Type IV Protein TRPML1
Source: PLoS One. 2013 Feb 13;8(2):e56780. doi: 10.1371/journal.pone.0056780 (PMC3572064; doi:10.1371/journal.pone.0056780)
Supplement: Table S3 — List of proteins identified by Split Ubiquitin Yeast Two-HybridScreens. (DOCX) [file pone.0056780.s003.docx]

| **Supplemental Table S3. Split-Ubiquitin Yeast Two-Hybrid Screen Data** | | | |
| --- | --- | --- | --- |
| Protein | NCBI Number | NubG fused to | No. of Independent Isolates |
| ERGIC and Golgi 3 (ERGIC) | NP_079792 | COOH-terminus | 1 |
| Phosphatidylinositol 4-phosphate 5-kinase type I-beta (P5KT1) | BAA13031 | COOH-terminus | 1 |
| Yip1 interacting factor homolog B isoform 2 (YIF1) | NP_001103671 | COOH-terminus | 1 |
| Protocadherin beta 20 | NP_444375 | COOH-terminus | 1 |
| Protocadherin gamma subfamily A2 | NP_291063 | COOH-terminus | 1 |
| Similar to Glyceraldehyde-3-phosphate dehydrogenase isoform 3 | [XP_001479421.2](http://www.ncbi.nlm.nih.gov/protein/309266468?report=genbank&log$=prottop&blast_rank=1&RID=AJY4RHM5013) | COOH-terminus | 1 |
| Voltage-gated sodium channel type V alpha | NP_067519 | COOH-terminus | 2 |
| FERM domain containing 5 (Frmd5) | CAQ12956 | COOH-terminus | 1 |
| Retrovirus-related Env polyprotein from Fv-4 locus | P11370 | COOH-terminus | 1 |
| Vacuolar protein sorting 4b (VPS4b) | NP_033216 | COOH-terminus | 1 |
| Btbd2 protein | AAI15684 | COOH-terminus | 1 |
| Open reading frame 61, isoform CRA_c (Membralin) | EDL31617 | COOH-terminus | 1 |
| Transmembrane Protein 163 (Cation Efflux Motif) | NP_082411 | COOH-terminus | 1 |
| Runt-related transcription factor 2 | EDL23422 | COOH-terminus | 1 |
| Ubc protein | AAH25894 | COOH-terminus | 1 |
| Gja1 protein | AAH55375 | COOH-terminus | 1 |
| ATPase, Ca^++^ transporting, slow twitch 2 isoform b | NP_033852 | COOH-terminus | 11 |
| mCG10343, isoform CRA_b (mitochondrial carrier protein) | EDL21527 | COOH-terminus | 1 |
| Retinoic acid induced 17, isoform CRA_d | EAW54643 | COOH-terminus | 1 |
| mCG133388, isoform CRA_e | EDL10122 | COOH-terminus | 2 |
| RIKEN cDNA 3110045G13, isoform CRA_b | EDL15349 | COOH-terminus | 1 |
| mCG10343, isoform CRA_b (mitochondrial carrier protein) | EDL21527 | COOH-terminus | 1 |
| mCG127133, isoform CRA_c | EDK97851 | COOH-terminus | 1 |
| Unnamed protein | BAE21227 | COOH-terminus | 1 |
| mKIAA0620 protein (Plexin) | BAC97985 | COOH-terminus | 1 |
| Zmiz1 protein | AAH58646 | COOH-terminus | 1 |
| Clk2-Scamp3 protein | ACC61069 | COOH-terminus | 1 |
| Unnamed protein (BAE) | BAE30441 | NH_2_-terminus | 1 |
| Peroxisomal membrane protein 2 (PMP2) | NP_033019 | NH_2_-terminus | 2 |
| Peroxisomal biogenesis factor 16 (PEX16) | Q91XC9 | NH_2_-terminus | 1 |
| Surfeit gene 4 | NP_035642 | NH_2_-terminus | 1 |
| Lysosomal-associated protein transmembrane 4B | NP_277056 | NH_2_-terminus | 1 |
| ATP synthase F0 subunit 6 | YP_002381175 | NH_2_-terminus | 1 |
| NADH dehydrogenase subunit 1 | YP_002791042 | NH_2_-terminus | 4 |
| NADH dehydrogenase subunit 5 | AAY96699 | NH_2_-terminus | 2 |
| This is the list of proteins that were isolated in split ubiquitin yeast two-hybrid screens with TRPML1-Cub-LexA-VP16. Rows highlighted in yellow are proteins that were retested in this study. Homologous proteins found by Split-Ubiquitin Yeast Two-Hybrid (this table) and by Mass Spectrometry (Supplementary Table 2) and are highlighted with matching colors. | | | |
